# Prevalence of genetic differences in phosphorylcholine expression between nontypeable *Haemophilus influenzae *and *Haemophilus haemolyticus*

**DOI:** 10.1186/1471-2180-10-286

**Published:** 2010-11-12

**Authors:** Kirk W McCrea, Jingping Xie, Carl F Marrs, Janet R Gilsdorf

**Affiliations:** 1 Department of Pediatrics and Communicable Diseases, University of Michigan, Ann Arbor, MI 48109, USA; 2 Department of Epidemiology, University of Michigan, Ann Arbor, MI 48109-0244, USA; 3 Beacon Analytical Systems Inc., Saco, ME 04072, USA

## Abstract

**Background:**

Although non-typeable (NT) *Haemophilus influenzae *and *Haemophilus haemolyticus *are closely related human commensals, *H. haemolyticus *is non-pathogenic while NT *H. influenzae *is an important cause of respiratory tract infections. Phase-variable phosphorylcholine (ChoP) modification of lipooligosaccharide (LOS) is a NT *H. influenzae *virulence factor that, paradoxically, may also promote complement activation by binding C-reactive protein (CRP). CRP is known to bind more to ChoP positioned distally than proximally in LOS, and the position of ChoP within LOS is dictated by specific *licD *alleles (designated here as *licD_I_*, *licD_III_*, and *licD_IV_*) that are present in a *lic1 *locus. The *lic1 *locus contains the *licA*-*licD *genes, and ChoP-host interactions may also be influenced by a second *lic1 *locus that allows for dual ChoP substitutions in the same strain, or by the number of *licA *gene tetranucleotide repeats (5'-CAAT-3') that reflect phase-variation mutation rates.

**Results:**

Using dot-blot hybridization, 92% of 88 NT *H. influenzae *and 42.6% of 109 *H. haemolyticus *strains possessed a *lic1 *locus. Eight percent of NT *H. influenzae *and none of the *H. haemolyticus *strains possessed dual copies of *lic1*. The *licD_III _*and *licD_IV _*gene alleles were distributed similarly (18-22%) among the NT *H. influenzae *and *H. haemolyticus *strains while *licD_I _*alleles were present in 45.5% of NT *H. influenzae *but in less than 1% of *H. haemolyticus *strains (*P *< .0001). NT *H. influenzae *had an average of 26.8 tetranucleotide repeats in *licA *compared to14.8 repeats in *H. haemolyticus *(*P *< .05). In addition, NT *H. influenzae *strains that possessed a *licD_III _*allele had increased numbers of repeats compared to NT *H. influenzae *with other *licD *alleles (*P *< .05).

**Conclusions:**

These data demonstrate that genetic similarities and differences of ChoP expression exist between NT *H. influenzae *and *H. haemolyticus *and strengthen the hypothesis that, at the population level, these differences may, in part, provide an advantage in the virulence of NT *H. influenzae*.

## Background

Strains of non-typeable (NT) *Haemophilus influenzae *asymptomatically colonize the human pharynx, but are also opportunistic pathogens that cause localized respiratory tract infections such as otitis media, pneumonia, bronchitis, sinusitis, and COPD exacerbation [1,2]. Bacterial factors that differentiate disease from commensal strains are largely unknown since the population structure of NT *H. influenzae *is genetically heterologous [3]. The association of bacterial factors with disease-causing strains can be inferred, however, by comparing the prevalence of genetic traits between epidemiologically defined collections of disease and commensal strains [4-7] or, alternatively, between the pathogenic species and a phylogenetically close but non-pathogenic relative [8-11].

*Haemophilus haemolyticus *is a phylogenetically close relative of NT *H. influenzae*, but has not been associated with disease [7,12,13]. The two species reside in the same host niche, overlap extensively by both taxonomic and phylogenetic analyses [10,14,15], and exchange DNA through natural transformation [10,13,16]. Given their close relationship, but difference in disease potential, NT *H. influenzae *and *H. haemolyticus *likely possess common genes or genetic traits for commensal growth but differ in genes or traits that facilitate disease [10].

Historically, *H. haemolyticus *has been considered a rarely encountered commensal that was easily differentiated from NT *H. influenzae *by its hemolytic phenotype [17-19]. Recent studies, however, have shown that 20-40% of isolates in various NT *H. influenzae *collections were miss-classified, and found to be non-hemolytic *H. haemolyticus *[7,13]. These observations suggest that *H. haemolyticus *is significantly more prevalent in the pharynges than previously thought, and that clinical differentiation of the species from throat and sputum samples is inadequate [13]. Therefore, we recently sought to differentiate the species by their relative proportions of selected NT *H. influenzae *virulence genes and observed that a probe made to *licA*, a NT *H. influenzae *gene necessary for phosphorylcholine (ChoP) modification of LOS, hybridized to 96% of NT *H. influenzae *isolates and to 42% of *H. haemolyticus *isolates [10]. The relationship of ChoP expression between NT *H. influenzae *and *H. haemolyticus *is unknown but differences between the species may highlight important roles in NT *H. influenzae *virulence.

In studies addressing NT *H. influenzae *virulence, ChoP-modified LOS has been shown to promote bacterial adherence and invasion of host cells through interaction with the platelet activating factor receptor, to increase bacterial resistance to host antimicrobial peptides such as cathelicidin (or LL-37/hCAP18), and to modulate the host inflammatory response directed toward bacteria present in biofilms [20-22]. Paradoxical to its role in enhancing colonization and virulence, ChoP can bind C-reactive protein (CRP) which initiates C1q binding that leads to activation of the classical complement pathway and bactericidal killing [23]. The concentration of CRP (in both serum and respiratory tract secretions) dramatically increases during inflammation, and has been proposed to facilitate clearance of ChoP-expressing bacteria in the respiratory tract [24,25]. Human ChoP-specific antibodies capable of eliciting in vitro bactericidal activity against some *H. influenzae *strains have also been identified, suggesting a further liability of *H. influenzae *ChoP expression [26]. *H. influenzae *may avoid CRP and anti-ChoP antibody binding, however, by phase varying ChoP expression and by strain-dependent localization of ChoP substitutions within LOS [27,28].

In *H. influenzae*, ChoP expression is controlled by a contingency locus, *lic1*, that contains the *licA*, *licB*, *licC*, and *licD *genes (encoding a choline kinase, a choline permease, a pyrophosphorylase, and a diphosphonucleoside choline transferase, respectively) [29]. Contingency loci, such as *lic1*, contain simple sequence repeats (SSR) that provide an organism with the ability to phase vary specific phenotypes in response to host challenges [27]. In *lic1*, the SSR are tetranucleotide (5'-CAAT-3') and are present at the 5' end of *licA*, the first gene in the locus [29]. During replication, intragenic SSR repeats undergo slipped-strand mispairing which results in translational phase variation, and the rate of these mutations is proportional to the length of the repeat region [30]. De Bolle et al [31] found that mutation rates of a *H. influenzae *type III restriction modification gene (*mod*) engineered to contain 17-38 tetranucleotide (AGTC) intragenic repeats increased linearly with the number of repeats. In contrast, the same gene containing 5-11 repeats demonstrated rare, if any, phase-variation. Thus, higher numbers of repeats in a contingency locus may protect the bacteria by decreasing the response time to host challenges [27]. Among *H. influenzae *strains, however, the number of *licA *gene 5'-CAAT-3' repeats range from 3-56, and patterns pertaining to virulence have not been identified [32,33].

Depending on the *H. influenzae *strain, ChoP may be substituted at different positions within LOS. Substitutions may occur on oligosaccharides that extend from any one of the three conserved inner-core heptose residues (heptose I, II, and III) or, alternatively, directly to heptose IV, an outer core heptose that extends from heptose I [34,35]. These substitutions are dictated largely by the diphosphonucleoside choline transferase encoded by the *licD *gene. Three *licD *gene alleles mediate ChoP substitutions at different positions within LOS and, for simplification, we have named the alleles to reflect their association with a given heptose-residue: *licD_I_*, *licD_III_*, and *licD_IV_*. Although ChoP has been associated with heptose II residues in selected strains, a specific *licD *allele mediating these substitutions has not been experimentally documented [35]. The deduced LicD proteins are 265-268 amino acids in length and range in sequence identity from 74-88% with much of the variation occurring in the central part of the primary structure [28,35]. Although most NT *H. influenzae *strains possess a single *licD *allelic gene that facilitates one ChoP substitution, Fox et al [35] recently reported that 4/25 (16%) of NT *H. influenzae *middle ear strains possessed two different *licD *alleles, each present in a separate, phase-variable *lic1 *locus, that together could produce up to two ChoP substitutions in the strain's LOS.

Both the number and position of ChoP substitutions within LOS may affect binding of host clearance molecules such as CRP or natural ChoP antibodies [26,28]. For instance, *H. influenzae *strains with dual ChoP substitutions bind more CRP, and *H. influenzae *strains with ChoP substitutions positioned from the distal heptose III residue are 10-fold more sensitive to CRP-initiated bactericidal killing than ChoP associated with the proximal heptose I in the same strains [28,35]. Consequently, strains with proximal ChoP substitutions (i.e. heptose I) may be more protected from CRP-mediated clearance, and LOS structural studies on selected NT *H. influenzae *strains have found that ChoP predominate at this position [34]. The overall prevalence of these substitutions in the NT *H. influenzae *population, however, is not known. Differences in the prevalence of single or combined *licD *gene alleles between NT *H. influenzae *and *H. haemolyticus *may reflect the importance of ChoP structures in NT *H. influenzae *virulence.

The presence of a *licA *gene in *H. haemolyticus *suggests that it may contain a *lic1 *locus and express ChoP in a manner similar to *H. influenzae *[10]. Since ChoP expression among NT *H. influenzae *strains can vary greatly due to genetic factors listed above, we speculated that differences in the prevalence of these factors between strain populations of *H. influenzae *and *H. haemolyticus *may highlight, in part, which ones provide an advantage to *H. influenzae *in transcending from commensal to disease-related growth.

## Results

### ChoP expression in *H. haemolyticus*

**Figure 1:**
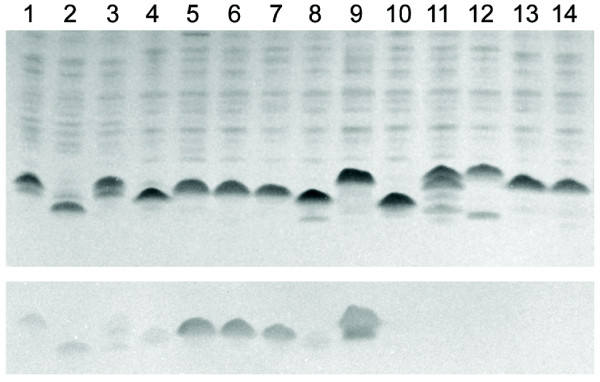
**LOS profiles and TEPC-15 mAb reactivity in *H. haemolyticus***. *H. influenzae *and *H. haemolyticus *whole-cell lysates were run on tricine SDS-PAGE and silver stained to visualize LOS migration (upper panel) or transferred to nitrocellulose membrane for reactivity with the ChoP-specific mAb, TEPC-15 (lower panel). Lanes 1-3, *H. influenzae *ChoP phase-on variant strains (E1a, Rd, and Mr15); lanes 4-9, *H. haemolyticus *strains hybridizing with a *licA *gene probe (M07-22, 60P3H1, 7P24 H, 3P41H5, C03-22, and H01-21); and lanes 10-14, *H. haemolyticus *strains not hybridizing with a *licA *gene probe (ATCC 33390, 3P18H1, 24P4 H, 26428, 26322)

Although *H. influenzae *is known to modify its LOS with ChoP, expression and surface localization of ChoP in *H. haemolyticus *has not been demonstrated. To investigate ChoP expression in *H. haemolyticus*, we obtained LOS profiles on silver-stained tricine SDS-PAGE from whole-cell lysates on three *H. influenzae *control strains, six *H. haemolyticus *strains containing a *licA *gene, and five *H. haemolyticus *strains lacking a *licA *gene [10]. As seen in Figure 1 (upper panel), both NT *H. influenzae *and *H. haemolyticus *demonstrated intra-and inter-strain variability in LOS migration. A duplicate gel was transferred to a Western immunoblot and ChoP was detected with TEPC-15, a mAb that recognizes ChoP on a number of pathogenic bacteria [36-38]. TEPC-15 reacted with LOS-associated bands in all *H. influenzae *control strains and in the six *H. haemolyticus *strains that contained a *licA *gene (Figure 1 lower panel). The antibody, however, did not react to five *H. haemolyticus *strains lacking a *licA *gene (Figure 1 lower panel).

The association of ChoP epitopes with *H. haemolyticus *LOS was further supported by proteinase K digestion experiments. TEPC-15 reactivity was still present on Western immunoblots containing *H. influenzae *strain Rd and *H. haemolyticus *strain M07-22 that were pre-treated with proteinase K, although no proteins were visible in these preparations when they were run on glycine SDS-PAGE and stained with Coomassie (data not shown). Together these results suggest that, similar to *H. influenzae*, some strains of *H. haemolyticus *can express a ChoP epitope that is localized within its LOS.

### *H. haemolyticus *contains a *lic1 *locus similar to *H. influenzae*

The ability of *H. haemolyticus *to hybridize with a *H. influenzae licA *gene probe suggests that *H. haemolyticus *contains a *lic1 *locus [10]. In *H. haemolyticus *strains M07-22 and 60P3H1, *licA*-*licD *gene probes were each found to hybridize with one restriction fragment on Southern blots, suggesting that all genes were confined to a single locus in each strain (data not shown). PCR designed to amplify overlapping regions of *H. influenzae lic1 *locus genes also amplified similar products in *H. haemolyticus *strains M07-22 and 60P3H1, and DNA sequencing of these products revealed that the strains contained tandem *licA-licD *genes spanning 3375 and 3324 bp [GenBank: HM140372 and HM140373], respectively, similar in size to the *lic1 *loci present in the genome-sequenced *H. influenzae *strains Rd (3358 bp) and 86-028NP (3333 bp) [39,40]. Further comparisons of the *lic1 *loci between *H. haemolyticus *and *H. influenzae *[29] revealed that, in both species, the loci were flanked by the same chromosomal genes, contained *licA *α, β, and γ start codons positioned immediately upstream of tandemly arranged tetranucleotide (5'-CAAT-3') repeats, and contained *licB *and *licC *start codons that overlapped each preceding gene (data not shown).

The LicA, LicB, and LicC amino-acid sequences for the two *H. haemolyticus *strains M07-22 and 60P3H1 were deduced and found to be 93, 99, and 95% identical, respectively, between the strains (Table 1). Amino-acid sequences comparisons of the putative LicA, LicB, and LicC proteins between *H. haemolyticus *and *H. influenzae *(strains E1a, Rd, and 86-028NP) revealed identities that were somewhat lower, ranging from 87-94% for all comparisons (Table 1). As mentioned above, three LicD protein alleles (LicD_I_, LicD_III_, and LicD_IV_) have been described for *H. influenzae*. The LicD protein of *H. haemolyticus *strain M07-22 was 89 and 87% identical to the LicD_I _allele of *H. influenzae *strains Rd and 86-028NP, respectively, but was 95% identical with and contained a 3 amino-acid insertion similar to the LicD_III _allele of *H. influenzae *strain E1a, suggesting that this *H. haemolyticus *strain possessed a LicD_III _allele (Table 1). In contrast, the putative LicD protein of *H. haemolyticus *strain 60P3H1 averaged only 69% identity with the LicD alleles of *H. haemolyticus *strain M07-22 and the three *H. influenzae *strains (Table 1). BLAST analysis, however, revealed that it was 99% identical to the deduced LicD_IV _protein of NT *H. influenzae *strain R2866, suggesting that *H. haemolyticus *strain 60P3H1 contained a LicD_IV _allele. Together, these data suggest that *H. haemolyticus *possess *lic1 *loci that are very similar to the *lic1 *loci described for *H. influenzae*.

**Table 1:** Amino-acid sequence identities between the LicA-LicD proteins of *H. influenzae *and *H. haemolyticus*

	LicA		LicB		LicC		LicD	
Strains	M07-22	60P3H1	M07-22	60P3H1	M07-22	60P3H1	M07-22	60P3H1
E1a	87.2	86.9	92.8	93.5	89.7	89.3	94.8	68.7
Rd	86.9	86.9	93.2	93.8	92.7	92.3	89.4	69.4
86-028NP	86.9	86.9	89.7	90.1	89.7	89.3	87.2	68.3
60P3H1	93.3		99.3		94.8		69.1	

### Prevalence of *lic1 *loci in *H. influenzae *and *H. haemolyticus*

As mentioned, the prevalence of the *licA *gene has been reported for a phylogenetically defined NT *H. influenzae *and *H. haemolyticus *strain collection [10]. We therefore determined the distribution of the remaining *lic1 *locus genes (*licB*, *licC*, and *licD*) among the same strains by dot-blot hybridization. The *licB*-*licD *gene probes each hybridized to three *H. influenzae *positive control strains (Rd, 86-028NP, and R2866), to 81/88 (92%) NT *H. influenzae *strains and to 46/109 (42.2%) *H. haemolyticus *strains. Four NT *H. influenzae *strains (53122, B01-21, H08-25, and H10-21) that previously hybridized with the *licA *gene probe did not hybridize with the *licB*-*licD *probes. In addition, one NT *H. influenzae *strain (32324) that did not previously hybridize with the *licA *gene probe did hybridize with the *licB*-*licD *probes in this study. Repeat hybridization of these discrepant strains with the *licA *gene probe revealed that *licA *hybridization was concordant with *licB*-*licD *hybridization, and that all strains either lacked or possessed all four *lic1 *locus genes. The probes did not hybridize to a negative control species (*N*. *meningitidis*) or to any of the remaining NT *H. influenzae *or *H. haemolyticus *strains that previously failed to hybridize with the *licA *gene probe (Table 2). The absence of the *licA*-*licD *genes in these strains suggests that 8% of NT *H. influenzae *and 57.8% of *H. haemolyticus *strains lack a *lic1 *locus for ChoP expression, and that absence of a *lic1 *locus is 7.23 times more prevalent in *H. haemolyticus *than in NT *H. influenzae *(expressed in Table 2 as 0.14 times prevalent for NT *H. influenzae*, *P *< .05).

**Table 2:** Prevalence of *lic1 *locus copy number and *licD *alleles in NT *H. influenzae *and *H. haemolyticus*

Genotype	*H. influenzae *n = 88 (%)	*H. haemolyticus *n = 109 (%)	PR^a^	*P *value^c^
*lic1 *copy number				
0	7 (8.0)	63 (57.8)	0.14	< .0001
1	74 (84.0)	46 (42.2)	2.18	< .0001
2	7 (8.0)	0 (0)^b^	ND	.0031
single *licD *alleles				
*licD_I_*	40 (45.5)	1 (0.92)	49.5	< .0001
*licD_III_*	14 (15.9)	23 (21.1)	0.75	.6647
*licD_IV_*	20 (22.7)	23 (21.1)	1.07	.3536
dual *licD *alleles				
*licD_IV_*-*licD_III_*	4 (4.5)	0 (0)	ND	.0383
*licD_I_*-*licD_III_*	1 (1.1)	0 (0)	ND	.4467
*licD_I_*-*licD_IV_*	1 (1.1)	0 (0)	ND	.4467
*licD_I_*-*licD_I_*	1 (1.1)	0 (0)	ND	.4467

^a ^Prevalence ratios (PR) were calculated for *H. influenzae *using *H. haemolyticus *as the referent group.

^b ^Logit, 0.5 used in place of 0 for PR and statistical calculations.

^c ^*P *< 0.05 is considered statistically significant using χ^2 ^analysis.

The prevalence of NT *H. influenzae *and *H. haemolyticus *strains possessing single or duplicate *lic1 *loci is not known. Similar to the method reported by Fox et al [35], we screened our 81 NT *H. influenzae *and 46 *H. haemolyticus lic1*-containing strains for duplicate *lic1 *loci using Southern hybridization of *Mfe*1 digested genomic DNA to identify two restriction fragments that hybridized with a *licD *gene probe. Strains with two *licD*-hybridizing bands were present in seven NT *H. influenzae *strains and in none of the *H. haemolyticus *strains. Further hybridization using a *licA *gene probe on the seven NT *H. influenzae *strains also revealed two *licA *hybridizing bands in these strains, suggesting that they possessed two complete *lic1 *loci. Assessing the population prevalence of *lic1 *locus copy number among the species, the data suggest that 74/88 (84%) NT *H. influenzae *and 46/109 (42.2%) *H. haemolyticus *possess one copy of *lic1*, and that strains with one *lic1 *locus are 2.18 times more prevalent in NT *H. influenzae *than in *H. haemolyticus *(*P *< .0001) (Table 2). Duplicate *lic1 *loci were present in 7/88 (8%) NT *H. influenzae *and 0/109 (0%) *H. haemolyticus *strains, suggesting that duplicate *lic1 *loci in *H. haemolyticus *are rare or altogether absent (Table 2).

### Prevalence of the three LicD alleles in NT *H. influenzae *and *H. haemolyticus*

**Figure 2:**
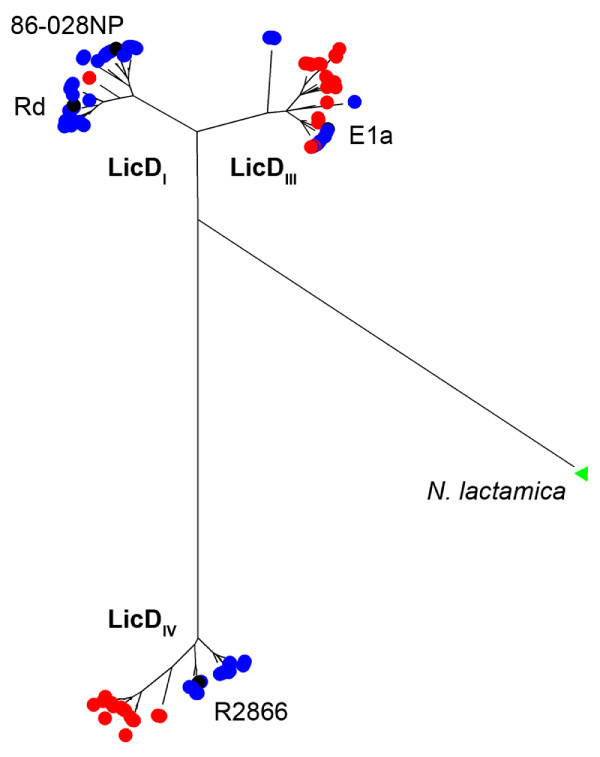
**Clustering of *H. influenzae *and *H. haemolyticus *LicD alleles**. The major clusters of *H. influenzae *(blue dots) and *H. haemolyticus *(red dots) strains are labeled by their predicted allele (LicD_I_, LicD_III_, and LicD_IV_) and prototype LicD alleles from *H. influenzae *strains are shown for each cluster (black dots, E1a is partially hidden). The LicD protein of *N*. *lactamica *is the out-group for the analysis (green triangle).

Determining the prevalence of the three previously described *licD *alleles among the two species was initiated by PCR amplification and DNA sequence analysis of the *licD *genes from the 74 NT *H. influenzae *and 46 *H. haemolyticus *strains in our collection that contained a single *lic1 *locus. The deduced LicD amino-acid sequences of these strains were determined [GenBank:HM133649-HM133768] and the *licD *gene from one NT *H. influenzae *strain (Mr27) was repeatedly found to possess a nonsense mutation that would result in gene termination. A minimum-evolution dendrogram (in radiation view) was created from the remaining LicD amino-acid sequences of the NT *H. influenzae *and *H. haemolyticus *strains. The dendrogram revealed three distinct clusters, each containing a different *H. influenzae *prototype LicD allele (LicD_I _from strains Rd and 86-023NP, LicD_III _from strain E1a, and LicD_IV _from strain R2866) (Figure 2). These results suggest that the three previously defined LicD alleles represent the major allelic variants found among the *H. influenzae *and *H. haemolyticus *species.

Next, we determined the population prevalence of specific *licD *alleles in our NT *H. influenzae *and *H. haemolyticus *strains. Among the 88 total NT *H. influenzae *strains in the collection, 43 (49%) possessed a single *licD_I _*allele, 19 (22%) possessed a single *licD_III _*allele, and 25 (28%) possessed a single *licD_IV _*allele (Table 2). In contrast, only 1 of the 109 (0.9%) *H. haemolyticus *strains possessed a *licD_I _*allele while 23 (21%) possessed a single *licD_III _*allele and 23 (21%) possessed a single *licD_IV _*allele. Although the prevalence of single *licD_I _*alleles was statistically different between NT *H. influenzae *and *H. haemolyticus *(*P *< .0001), the prevalence of the *licD_III _*and *licD_IV _*alleles was not statistically different between the species (Table 2). Assessment of *licD *gene alleles among the seven dual *lic1 *locus-containing NT *H. influenzae *strains was determined by PCR amplifying and sequencing *licD *from agarose gel slices of strain DNA digested with *Mfe*1. The results revealed that 4/88 (4.5%) strains had *licD_III_*-*licD_IV _*alleles, while only 1/88 (1.1%) strains each were found to possess combinations of *licD_I_*-*licD_III_*, *licD_I_*-*licD_IV_*, and *licD_I_*-*licD_I _*alleles (Table 2). Together, these results suggest that the *licD_I _*allele is rarely present in *H. haemolyticus*, and that the proportions of *licD_III _*and *licD_IV _*alleles are similar between the species.

### ChoP phase variation and the number of *licA *tetranucleotide (5'-CAAT-3') repeats among NT *H. influenzae *and *H. haemolyticus*

Phase variation of ChoP expression is similar between NT *H. influenzae *and *H. haemolyticus*. The *licA *genes of *H. haemolyticus *strains M07-22 and 60P3H1 contained a number of 5'-CAAT-3' repeats that would place the *licA *gene in a correct translational open reading frame (data not shown). ChoP expression in these two strains was corroborated by Western immunoblot where TEPC-15 reactive epitopes were present in each strain (Figure 1, lanes 4 and 5). In addition, phase-negative variants could be isolated from each *H. haemolyticus *strain, and DNA sequence analysis revealed that each *licA *repeat region gained one 5'-CAAT-3' repeat, placing the *licA *gene out of frame (data not shown).

**Figure 3:**
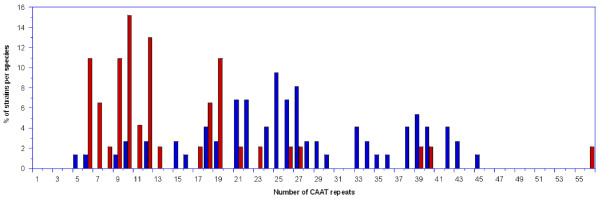
**Distribution of NT *H. influenzae *and *H. haemolyticus *strains with various numbers of CAAT repeats**. Percent of *lic1*-positive NT *H. influenzae *and *H. haemolyticus *strains based on the number of CAAT repeats they contain. NT *H. influenzae *and *H. haemolyticus *are labeled in blue and red, respectively.

Mutation rates in contingency loci are proportional to the length of the repeat region in the loci and the repeat region length may therefore affect the ability of bacteria to respond to a host immunologic challenge [31]. To determine if a general population difference of *licA *repeat length exists between the species in this study, we compared the number of *licA *5'-CAAT-3' repeats between the 74 NT *H. influenzae *and 46 *H. haemolyticus *strains that contained a single *lic1 *locus. DNA sequence analysis of PCR amplified repeat regions from these strains revealed a wide range in repeat numbers for both species (5-45 and 6-56 repeats for NT *H. influenzae *and *H. haemolyticus*, respectively) (Figure 3, Table 3). The average number of *licA *repeats between the species, however, was statistically different with NT *H. influenzae *having a mean of 27 repeats and *H. haemolyticus *having a mean of 15 repeats (*P *< .0001 using the student's T test) (Table 3). These results suggest that, at the population level, the contingency response for ChoP expression may be slower for *H. haemolyticus *than for NT *H. influenzae*.

**Table 3:** Stratification of the number of *licA *gene 5'-CAAT-3' repeats between species and *licD *alleles

Stratification	Strains (n)	Range	**Average ± S.D**.
Species			
NT *H. influenzae*	74	5-45	27 ± 10*
*H. haemolyticus*	46	6-56	15 ± 4
NT *H. influenzae licD *alleles			
*licD_I_*	40	6-45	25 ± 9
*licD_III_*	14	5-43	34 ± 11**
*licD_IV_*	20	9-42	26 ± 8
*H. haemolyticus licD *alleles			
*licD_III_*	23	6-56	16 ± 13
*licD_IV_*	23	6-27	13 ± 6

* *P *< .0001 using the student's T-test

** *P *< .05 for each comparison using the student's T-test

*H. influenzae *strains that express ChoP at more distal positions in LOS (i.e. ChoP substituted on an oligosaccharide extending from heptose III) have been shown to be more vulnerable to CRP binding, leading to activation of complement mediated bactericidal killing [28]. Therefore, strains may differ in their *licA *mutation rates depending on which LOS structure is modified with ChoP. To test this, we further stratified the number of *licA *gene repeats between strains with different *licD *alleles for each species. Among NT *H. influenzae*, the range of repeats was similar among strains that possessed a *licD*_I_, *licD_III_*, or *licD_IV _*allele (6-45, 5-43, and 9-42 repeats, respectively) (Table 3). The average number of repeats was significantly different, however, for strains that possessed a *licD_III _*allele (34 repeats) than for strains that possessed a *licD*_I _or *licD_IV _*allele (25 and 26 repeats, respectively) (*P *= .015 and .032 using the student's T test, respectively) (Table 3). Among *H. haemolyticus*, the range of *licA *repeats was more variable between strains with *licD_III _*and *licD_IV _*alleles (6-56 and 6-27 repeats, respectively), due mainly to three *licD_III_*-containing strains with *licA *genes that contained 39, 40, and 56 repeats (Table 3, Figure 3). In contrast to NT *H. influenzae*, however, the average number of repeats was not significantly different between *H. haemolyticus *strains possessing *licD_III _*or *licD_IV _*alleles (16 and 13, respectively) (Table 3). These results suggest that NT *H. influenzae *strains that substitute ChoP on more proximal, exposed oligosaccharides chains may tend to have increased mutation rates within the repeat region of the *licA *gene.

## Discussion

The strain population structure of NT *H. influenzae *is genetically very diverse and clones or clusters of NT *H. influenzae *strains that differentiate virulent from commensal strains have not been identified [10,41]. Given this diversity, together with the high prevalence of NT *H. influenzae *colonization in the healthy human population, it is reasonable to hypothesize that not all NT *H. influenzae *strains possess the same ability to cause disease, but rather, that a proportion of strains possess a range of variable genetic traits that allow for infection and disease under the right host conditions [42]. Thus, comparison of genetic trait prevalence between populations of NT *H. influenzae *and the closely related but strictly commensal species, *H. haemolyticus*, will highlight traits within the species' gene pools that may offer clues to the virulence pathways of NT *H. influenzae*. For instance, ChoP expression in NT *H. influenzae *is strongly implicated as a virulence factor [43,44] and is thought to enhance virulence though increased epithelial cell adherence, inhibition of bactericidal peptides, and modulation of the immune system during biofilm growth [20-22]. In this study, 58% of *H. haemolyticus *strains lacked a *lic1 *locus (and the ability to express ChoP) while only 8% of NT *H. influenzae *strains lacked a *lic1 *locus, suggesting that, at a population level, ChoP expression may provide an advantage for more NT *H. influenzae *strains to cause disease. Furthermore, the trend of shorter *licA *gene repeat regions in *H. haemolyticus *strains that possess a *lic1 *locus (and the potential to express ChoP), may suggest that those strains have a slower phase-variable response to host defences targeting ChoP (i.e. CRP), potentially limiting their survival in inflammatory environments. Obviously, prevalence differences in ChoP expression alone do not account for all differences in disease potential between the species since many other virulence factors have been described for NT *H. influenzae*. Rather, the differential prevalence of genetic traits between the species highlight factors that may be further studied for their roles in virulence using in vitro and in vivo models of NT *H. influenzae *infection.

Although the structure of *H. haemolyticus *LOS is unknown, the assumption has been made that basic LOS structures and biosynthesis of ChoP modifications, mediated by the phosphocholine transferase, LicD, are comparable between NT *H. influenzae *and *H. haemolyticus*. Some evidence suggests that these assumptions are reasonable. In the tricine SDS-PAGE experiments of this study, *H. haemolyticus *LOS migrated at a rate similar to the LOS of NT *H. influenzae*, and *H. haemolyticus *LOS also presented intra and inter-strain structural heterogeneity similar to the LOS of NT *H. influenzae *(Figure 1). Recent structural analysis on the LOS of *Haemophilus parainfluenzae*, a member of the *Pasteurellaceae *family that is phylogenetically more distant to NT *H. influenzae *than *H. haemolyticus*, revealed that the inner core structure was nearly identical to that of NT *H. influenzae *[45]. Furthermore, the LicD_III _and LicD_IV _alleles of the two *H. haemolyticus *strains in this study demonstrated higher sequence identity (95-99%) to their cognate proteins in NT *H. influenzae *than similar comparisons of LicA, LicB, and LicC proteins (87-94%, Table 1), suggesting a functional equivalence of the LicD protein alleles. Although these observations are circumstantial, they argue for more detailed comparisons of LOS structures between NT *H. influenzae *and *H. haemolyticus *to identify dissimilarities between the structures that may be associated with the ability of NT *H. influenzae *to cause disease.

The results of this study suggest that genotypes facilitating LOS-ChoP structures that are not conducive to CRP binding predominate among the strain populations of both species; the majority of *H. haemolyticus *strains (58%) lacked a *lic1 *locus (indicating no ChoP expression) and the majority of NT *H. influenzae *strains either lacked a *lic1 *locus or possessed a single *licD_I _*allele (an allele known to dampen CRP binding by positioning ChoP substitutions from the proximal inner core heptose) (54% total strains). In comparison, strains possessing single *licD_III _*and *licD_IV _*alleles were in smaller, but similar fractions in the strain populations of both species (16-23%), indicating that these allelic distributions are still maintained in the species despite possible increased vulnerability to CRP binding. Further studies that assess the prevalence of *licD *alleles between epidemiologically comparable collections of virulent and commensal NT *H. influenzae *strains may highlight which alleles are important in NT *H. influenzae *disease.

One ChoP genotype that may be associated with NT *H. influenzae *disease isolates is the possession of two *lic1 *loci in the same strain where each locus contains a different *licD *allele, providing the bacteria with two independently phase-variable ChoP substitutions. Fox et al [35] demonstrated that 4/25 (16%) NT *H. influenzae *middle ear strains had dual *lic1 *loci. In the current study, only NT *H. influenzae *and not *H. haemolyticus *possessed dual *lic1 *loci. Although only 7 of 88 (8%) total NT *H. influenzae *strains had dual loci, six were present among 43 (14%) middle ear strains present in this collection (unpublished results). Fox et al. [35] also noted that the genome sequenced NT *H. influenzae *strain, R2846, possessed a complete and partial *lic1 *loci, each containing a different *licD *allele, raising the possibility that other strains may have a similar genotype. An extensive search on the *lic1*-containing strains in this collection using *licD*-specific PCR and hybridization, however, did not identify any strains (apart from the seven dual *lic1 *locus strains) that contained more than one *licD *allele, suggesting that the NT *H. influenzae *population contains mainly complete copies of *lic1 *(unpublished results).

Although NT *H. influenzae *LOS structural studies have identified ChoP modifications on oligosaccharides extending from the heptose II position [46], specific *licD *alleles mediating this arrangement have not been characterized. It is possible that one or more of the current LicD alleles may overlap in this process or that stochastic factors in LOS biosynthesis may play a role. In addition, the clustering analysis of LicD protein alleles present in Figure 2 suggests that sub-variants may exist within the major allelic groups, and it is possible that one of these variants may facilitate heptose II-associated ChoP substitutions.

As reviewed by Moxon et al [27], strains that are genetically and epidemiologically unrelated vary widely in the lengths of SSR (including *licA *tetranucleotide repeats), while individual strains that transmit within an outbreak or are extensively subcultured over time maintain a central modality in repeat numbers [32,33]. Using a larger number of samples from a phylogenetically defined collection of NT *H. influenzae *strains has allowed us to partially resolve distribution trends for the *licA *repeat region in the NT *H. influenzae *and *H. haemolyticus *populations (Figure 3) and make statistical comparisons between and within species (Table 3). We found statistically significant trends toward the increased length of *licA *tetranucleotide repeats in NT *H. influenzae *compared to *H. haemolyticus*, and in NT *H. influenzae *strains with *licD_III _*alleles compared to NT *H. influenzae *strains with *licD*_I _or *licD_IV _*alleles. Longer repeat regions are predicted to increase *lic1 *loci mutation rates and ChoP phase variation, providing increased resistance to host clearance mechanisms such as CRP or antibodies that bind ChoP and initiate complement mediated bactericidal killing. The presence of the longest repeat (56 repeats) in a *H. haemolyticus *strain and only five repeats in a *licD_III_*-containing NT *H. influenzae *strain, however, are reminders that these trends must be considered in the light of numerous other factors that contribute to the commensal life style of both species and disease potential of NT *H. influenzae*.

## Conclusions

In summary, the *lic1 *locus is not part of the conserved "core" genome of the *H. influenzae *population but is part of the flexible gene pool that exists among different strains [47]. Nonetheless, the conserved chemical nature of ChoP and the discovery of anti-ChoP antibodies in human serum provides reasonable credence to ChoP as a vaccine candidate that may inhibit *H. influenzae *at some point in the infectious process. Knowledge of how ChoP expression varies both genetically and structurally within the NT *H. influenzae *strain population is critical for designing intervention strategies that will effectively target disease-related strains. Furthermore, contrasting the genetic properties of NT *H. influenzae *ChoP expression with those of *H. haemolyticus*, a closely related but non-pathogenic species, has highlighted a number of ChoP expression differences (*lic1 *copy number, *licD *alleles, and *licA *repeat number) that may provide an advantage to disease-related growth in NT *H. influenzae*.

## Methods

### Bacterial strains and culture methods

For most studies, bacteria were grown on chocolate agar plates (BBL). ChoP expression was carried out on Levinthal agar [48]. All cultures were incubated at 37°C with 5% CO_2_.

The 88 NT *H. influenzae *and 109 *H. haemolyticus *strains were parts of various collections obtained by this or other laboratories in previous studies [13,49-54] . All clinical and commensal strains in the current study were used with the approval of the University of Michigan Institutional Review Board. These same strains have been previously characterized for their taxonomic and phylogenetic relationships [10]. Reference strains used in this study included the complete or partially genome sequenced *H. influenzae *strains Rd (KW-20, ATCC 51907), 86-028NP [NT nasopharyngeal strain associated with otitis media], R2866 (INT-1, ATCC 51997; a NT, invasive strain), and a *H. haemolyticus *type strain, ATCC 33390. A negative-control species, *N*. *meningitidis *strain G1723, was used in dot-blot hybridization. Two *H. haemolyticus *strains, M07-22 and 60P3H1, were used to detail the *lic1 *locus and demonstrate ChoP expression in *H. haemolyticus*. M07-22 is a hemolytic strain obtained from the throat of a healthy child attending day care and 60P3H1 is a non-hemolytic strain from the sputum of an adult with COPD (although not associated with COPD exacerbation) [10,13].

### DNA isolation and PCR

Purified genomic DNA for Southern blots or PCR template was obtained from bacterial strains using the Wizard Genomic DNA purification kit from Promega, Co. (Madison, WI). Oligonucleotides for PCR amplification of gene probes, *lic1 *loci, and *licD *alleles were synthesized by Invitrogen and are shown in Table 4. PCR amplification of the tetranucleotide repeat region was performed as previously described [23] and sequence analysis was done with the primers listed in Table 4. PCR conditions have been described elsewhere [10] and all amplification products were confirmed by 1%-agarose gel electrophoresis.

**Table 4:** Oligonucleotides used in PCR or for DNA sequence analysis

Gene	Primer sequences^a^	Relative position in Rd	Use
*licA*	F^b^: GTAGGATTTGTTAAAACTTGCTACAAGCC	1608693	probe
	R: GGCAATTCCTCTAACAGTTTAAATGCTGCG	1609579	
*licA*	5'F1: GAATAAATTCATAAGAYTCAGAGCCTTAC	1608523	*lic1 *locus
	5'F2: CAGCTAACCGAGCTTGGGTGAGAAAGTGG	1608476	and
	mid R: GGCGAAACTCATCGAATACGC	1609107	5'-CAAT-
	3'R: GCCCAAAATACAGCGGACAG	1609626	3'
*licB*	F: ATGCGTGGCTATCTCTTTGGCATAC	1609583	probe
	R: TCATTTTTGTTCCCCTTTGTAATAAAGTG	1610461	
*licB*	5'F: GTTATTTGATATAGCGACGATCATTGAGG	1609316	*lic1 *locus
	mid F: CGGATTCGCCTTGGCTATTATTTCTTCTTCG	1609957	
	mid R: GAGGATATCACTATTTCAGATGACCACCC	1610091	
	3'R: GTGTAAATACCCTGTAACAATGACAATATTATCG	1610628	
*licC*	F: ATGAATGCAATCATTTTAGCAGCAGG	1610458	probe
	R: ATGTGGTGATAGTCATCAAGGTTATCC	1611125	
*licC*	mid F: CGTATTGATATTGGTTCACTGAATCAACCC	1610884	*lic1 *locus
*licD*	F: ATGAAAAAATTGACTCTCAGAG	1611159	probe
	R: TTACAAAATATACGCTTCTTGAATATG	1611956	
*licD*	5'F: AATTGGGATACCATTCCGATGG	1611016	*lic1 *locus
	3'R: AAGGGGCGCAAGAGCAGTTAG	1612129	and *licD *alleles

^a ^All oligonucleotides based on DNA sequences from *H. influenzae *strain Rd or from *H. haemolyticus lic1 *sequence in this paper to make dot-blot hybridization probes or sequence the *lic1 *locus, the *licD *gene alleles, or the *licA *gene tetranucleotide repeats

^b ^Forward primer begin downstream of *licA *gene tetranucleotide repeats

### DNA sequencing

DNA sequences of the *lic1 *loci of *H. haemolyticus *strains M07-22 and 60P3H1, the *licD *allelic genes and the tetranucleotide repeat regions of all strains in the collection possessing *licA*-*licD *genes were obtained from PCR products purified on QIAquick columns from Qiagen (Valencia, CA). Automated fluorescent dideoxy-DNA sequencing was done by the University of Michigan DNA sequencing core on an ABI model 3730 sequencer. Sequence editing and gene and locus assembly were done with Lasergene software (version 7.0; DNAStar, Madison, WI). Cluster analysis of the LicD protein alleles was done using Mega software (version 3.1) [55]. A bootstrap consensus, minimum-evolution dendrogram of LicD amino-acid sequence was made with 1,000 replicates.

### Dot and Southern-blot hybridization

The bacteria were harvested in PBS to an O.D._600 _of 1.0, lysed, and frozen as previously described [10]. For dot-blot analysis, 40 μl of crude lysate DNA obtained from *Haemophilus *strains grown on chocolate agar was applied in an 8 × 12 array on nylon membranes as previously described [10].

PCR-amplified genes were purified from agarose gels using the QIAquick Gel Extraction Kit (Qiagen), and labeled with the AlkPhos Direct™ Labeling and Detection System (GE Healthcare, Piscataway, NJ). Probes were hybridized to the dot-blot membranes under stringent conditions and developed by the ECF detection system (GE Healthcare). Probe signal intensity was read by a Storm™ 860 phosphorimager and analyzed with ImageQuant version 5.0 software (Molecular Dynamics/GE Healthcare) [10].

Southern blots to identify *lic1 *loci in *H. haemolyticus *strains M07-22 and 60P3H1 or to determine the prevalence of *lic1 *locus duplication in all strains with *licA*-*licD *genes contained purified strain DNA digested with *Eco*RI and *Mfe*1, respectively. As previously reported by Fox et al [35], strains with duplicate *lic1 *loci appear on Southern blots as two *Mfe*1 fragments that hybridize with either *licA *or *licD *gene probes. In our study, we used a *licD *gene probe consisting of combined PCR products representing all three *licD *alleles (*licD_I _*from NT *H. influenzae *strain 86-028NP and *licD_III _*and *licD_IV _*from *H. haemolyticus *strains M07-22 and 60P3H1, respectively). All gene probes were labeled, hybridized, and detected as described for dot-blot hybridization, above.

### SDS-PAGE and immunoassays

Whole-cell lysates for SDS-PAGE and Western blotting were obtained by harvesting bacteria in PBS to an O.D. of 1.0, and diluting 4 fold in tricine sample buffer. In the proteinase K experiments, 10 μl of the suspension was incubated with .5 mg/ml of proteinase K at 55 °C for 2 hours. Untreated or treated bacterial suspension and equal volumes of sample buffer were then heated at 100 °C for 10 min. and 3 μl of preparation were loaded and run on Novex 16% tricine SDS-PAGE gels and XCell *Surelock*™Mini-Cell apparatus (Invitrogen, Carlsbad, CA) according to the manufacturer's recommendations. Western transfer was performed on a Mini trans-blot apparatus from Bio-Rad on nitrocellulose membrane (NCM) from Millipore (Bedford, MA). Colony blots were prepared by suspending one colony from the strain of interest in 1 ml of PBS, and plating 100 μl of 10^-6 ^and 10^-8 ^dilutions on Levinthal agar. Following overnight growth, the colonies were blotted onto NCM discs (Millipore), and the blots were immediately washed in PBS and immunoassayed.

Western and colony-blot immunoassays were performed by first blocking membranes in PBS containing 2% non-fat dry milk [blotto [56]] for one hour. The blots were then placed in TEPC-15 mAb (Sigma) diluted 1:5000 in blotto for one hour, washed three times with PBS and incubated for one hour in PBS containing 1:5000 goat, anti-mouse IgA antibody conjugated to alkaline phosphatase (Sigma). Following three washes with PBS, a colorimetric reaction was obtained with nitroblue tetrazolium chloride (NBT)/5-bromo-4-chloro-3'-indolyphosphate p-toluidine salt (BCIP) substrate (Pierce, Rockford, IL).

### Statistical analyses

All prevalence data were entered in Excel software (Microsoft) in binary form for the presence (which was given a value of 1) or absence (which was given a value of 0) of any given ChoP-associated genotype. The prevalence ratios of genotypes between NT *H. influenzae *and *H. haemolyticus *were calculated as a ratio of the proportions of genotypes among each species. Chi-square analysis was used to determine the significance of the differences of the genotype associations between species. Statistical analyses were performed with SAS software (version 9.1). Statistical differences in the length of repeat-regions were tested by pair-wise comparisons with the student's T test.

## Authors' contributions

KWM conceived and directed the study design, performed genetic and immunologic assays, and wrote the manuscript. JX performed genetic assays and did the statistical analyses. CFM and JRG helped in the study design and draft of the manuscript. All authors read and approved the final manuscript.
